# Distal femoral osteotomy versus lateral unicompartmental arthroplasty for isolated lateral tibiofemoral osteoarthritis with intra-articular and extra-articular deformity: a propensity score-matched analysis

**DOI:** 10.1186/s43019-022-00164-0

**Published:** 2022-07-18

**Authors:** Gianluca Piovan, Luca Farinelli, Daniele Screpis, Venanzio Iacono, Lorenzo Povegliano, Marco Bonomo, Ludovica Auregli, Claudio Zorzi

**Affiliations:** 1 Department of Orthopaedics, IRCCS Ospedale Sacro Cuore Don Calabria, Negrar di Valpolicella, Italy; 2 Clinical Orthopaedics, Department of Clinical and Molecular Sciences, Università Politecnica delle Marche, Ancona, Italy

**Keywords:** Opening-wedge distal femoral osteotomy, Lateral unicompartmental arthroplasty, Lateral knee osteoarthritis, Middle-aged patients

## Abstract

**Purpose:**

Lateral unicompartmental arthroplasty (UKA) and distal femoral osteotomy (DFO) represent surgical solutions in cases of valgus malalignment and isolated lateral osteoarthritis (OA) of the knee. The aim of the present study was to assess the clinical results, complications, and the overall postoperative alignment of a series of DFO and lateral UKA with a minimum 2-year follow-up in active middle-aged patients.

**Methods:**

Patients with valgus knee and isolated lateral OA who underwent opening-wedge DFO or UKA from 2017 to 2019 were reviewed. Each patient was characterized by a joint line convergence angle (JLCA) > 3° and mechanical lateral distal femoral angle (mLDFA) < 87°. We excluded patients who underwent meniscus or osteochondral allograft during DFO. The Oxford Knee Score (OKS), Knee Injury and Osteoarthritis Outcome Score (KOOS), complications, and postoperative alignment were assessed. Propensity score matching was used to identify comparable patients.

**Results:**

The DFO and lateral UKA groups consisted of 29 patients each. No statistically significant differences in gender, age, body mass index (BMI), length of follow-up, or limb deformity were reported between the two groups. In the DFO group, OKS was reported to improve from 27.51 to 38.59 (*p* < 0.05) and KOOS from 51.14 to 67.2 (*p* < 0.05). Similarly, in the UKA group, OKS improved from 26.23 to 35.43 (*p* < 0.05) and KOOS from 50.12 to 65.91 (*p* < 0.05). However, the improvement in OKS and KOOS (delta) did not differ between groups (*p* = 0.35 and *p* = 0.95). The DFO and UKA groups were characterized by similar postoperative hip-knee-ankle (HKA) angle measurements of −3.26 and −3.00, respectively (*p* = 0.65). No patients in the UKA group underwent revision or other knee surgeries during follow-up. No infections were detected in either group. In the DFO group, no cases of nonunion or delayed union were reported. However, 40% of DFO patients underwent plate removal. One patient in each group was characterized by progression of medial OA with Kellgren-Lawrence (KL) grade > 3.

**Conclusion:**

UKA and DFO represent an effective treatment in lateral knee OA with intra-articular and extra-articular deformity. Both surgeries were able to provide a significant and comparable clinical improvement.

*Level of evidence*: III, comparative retrospective cohort study.

## Introduction

The treatment of symptomatic lateral knee osteoarthritis (OA) in young patients with valgus knee alignment is challenging. Total knee replacement (TKA), unicompartmental arthroplasty (UKA), and distal femur osteotomy (DFO) could be useful options [1]. It has been established that UKA and DFO are both indicated in the presence of valgus and isolated unicompartmental osteoarthritis in the absence of inflammatory arthritis, severe ligamentous instability, flexion contracture, and limited preoperative range of motion [2–6].

Traditionally, osteotomy is preferred in patients with constitutional extra-articular deformity who have active jobs or lifestyles and are under 60 years of age [7]. As concerns the grade of OA, no unique cutoff for cartilage damage indicating DFO has been reported in the literature. Indeed, a significant clinical improvement has also been reported for patients with late-stage OA [8]. Conversely, UKA is historically reserved for patients with lateral OA and intra-articular deformity, with a sedentary lifestyle and age greater than 60 years [9]. However, thanks to its modern design, favorable results have been published on the use of UKA in active young patients [10–12].

In cases of valgus knee and advanced lateral tibiofemoral OA with intra-articular and extra-articular deformity, the choice between UKA and DFO is controversial because their indications are comparable.

The purpose of the present study was to report the clinical results and complications of a series of DFO and lateral UKA with minimum 2-year follow-up in active middle-aged patients with advanced lateral tibiofemoral OA and intra-articular and extra-articular deformity. The null hypothesis was that DFO would offer similar clinical improvement compared with UKA in this selected population.

## Materials and methods

Data for patients who underwent opening-wedge DFO and lateral UKA for symptomatic lateral knee OA in valgus knee from 2017 to 2019 were retrospectively reviewed in June 2021. Data were prospectively collected and then reviewed. We designated two groups: opening-wedge DFO and lateral UKA. Subsequently, the following inclusion and exclusion criteria were applied in each group to obtain two cohorts of patients where indications of arthroplasty and preservation surgery were comparable [11, 13]. We included patients who met the following criteria: lateral knee OA defined as Kellgren–Lawrence (KL) grade III–IV; aged 45–60 years; body mass index (BMI) < 30; Tegner activity level > 4; joint line convergence angle (J LCA) > 3° and mechanical lateral distal femoral angle (mLDFA) < 87°; minimum 2 years of follow-up.

**Fig. 1:**
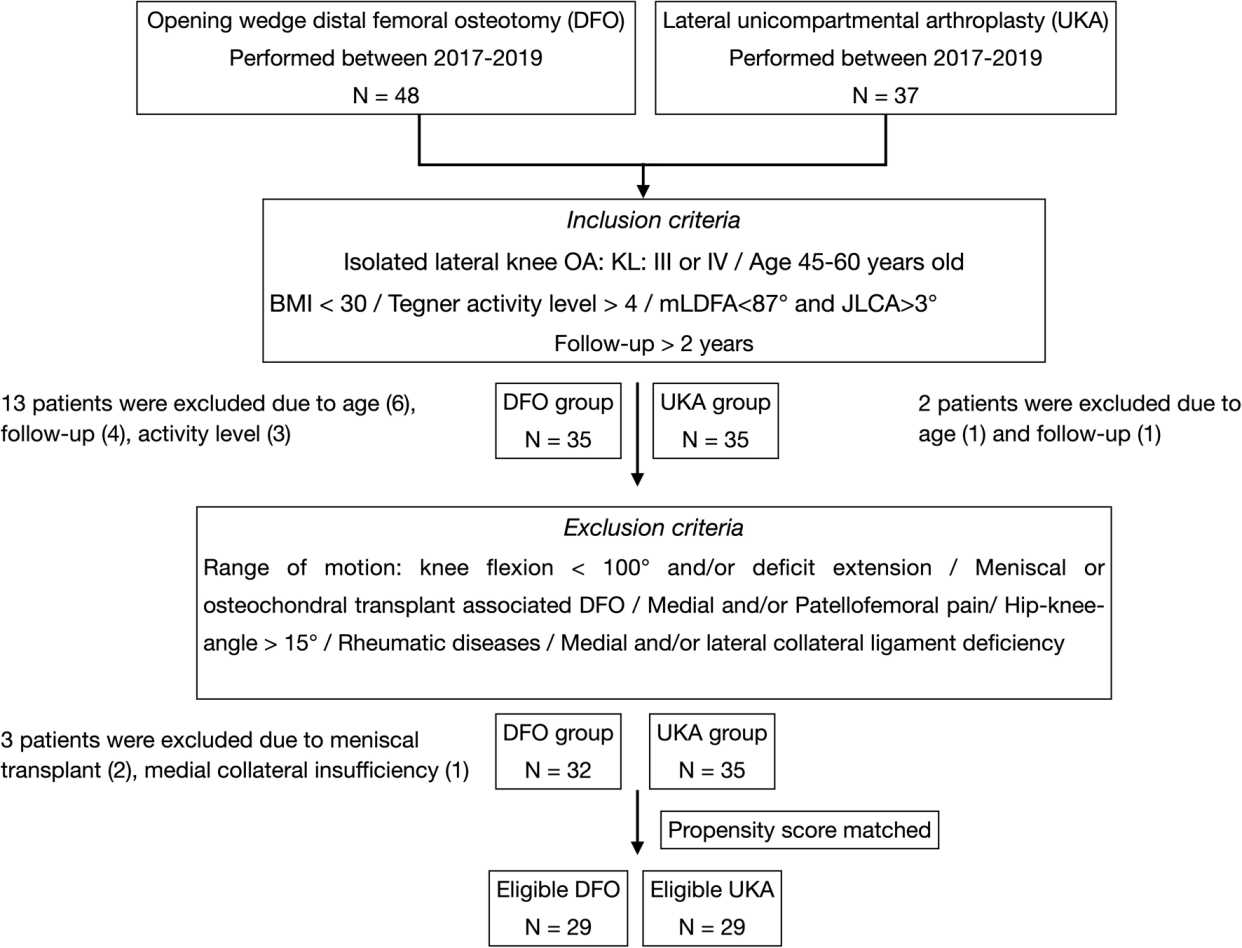
Inclusion and exclusion criteria. *BMI* body mass index, *mLDFA* mechanical lateral distal femoral angle, *JLCA* joint line convergence angle

We excluded patients who underwent associated procedures during DFO such as meniscus or osteochondral allograft transplant. Patients with preoperative valgus alignment of the lower limb with a hip-knee-ankle (HKA) angle greater than 15° were excluded (Fig. 1).

Considering the physical examination, we excluded patients with less than 100° knee flexion, and/or with knee extension deficit. The clinical evaluation of the patellofemoral and medial tibiofemoral joints is crucial in cases of DFO or lateral UKA. Therefore, we excluded patients with tenderness over the medial joint line and over the medial or lateral patellar facet [15]. We also excluded patients with lateral and/or medial collateral ligament deficiency.

Patients were clinically tested by senior surgeons (GP and CZ). Additionally, patients of both groups underwent magnetic resonance imaging (MRI) of the knee to exclude collateral ligaments and central pivot injuries. Anteroposterior, lateral-lateral, and Rosenberg radiographs were performed before surgery.

Each eligible patient was contacted and asked to participate in the study; at the follow-up evaluation, all patient signed an informed consent form. The study followed the current national and international laws and regulations governing the use of human subjects (Declaration of Helsinki and later amendments) and was approved by the local institutional review board (IRB). The Oxford Knee Score (OKS) and the Knee Injury and Osteoarthritis Outcome Score (KOOS) were used at the basal evaluation and recorded at follow-up. Age, gender, body mass index (BMI), analysis of the deformity in accordance with Paley [16], preoperative and follow-up long-leg standing radiograph, any complication related to surgery, and any other knee surgery on the ipsilateral side were noted from the medical chart.

### Preoperative assessment, surgical technique, and postoperative rehabilitation protocol

All surgeries in both the UKA and DFO groups were performed by the same surgical team (PG, SD, IV, CZ) with a high volume of knee procedures per year. Preoperative radiographic evaluation included a long-leg standing radiograph, standing anteroposterior and lateral views, standing posterior-anterior radiograph of both knees at 45° knee flexion (Rosenberg view), and patellar axial view at 30° knee flexion. Both groups of patients had undergone preoperative magnetic resonance imaging (MRI) to exclude lesions of the anterior cruciate ligament (ACL) or cartilage wear of the medial tibiofemoral or patellofemoral compartments not detectable on radiographs. An accurate physical examination was performed before and after anesthesia in order to detect any ligamentous instability, flexion deformity, or limited range of motion that could modify the surgical indication. The same perioperative protocol was followed for all patients in both groups. All patients received antibiotic prophylaxis with first-generation cephalosporin for the first 24 h postoperatively and thromboprophylaxis with low-molecular-weight heparin for 30 days postoperatively.

**Fig. 2:**
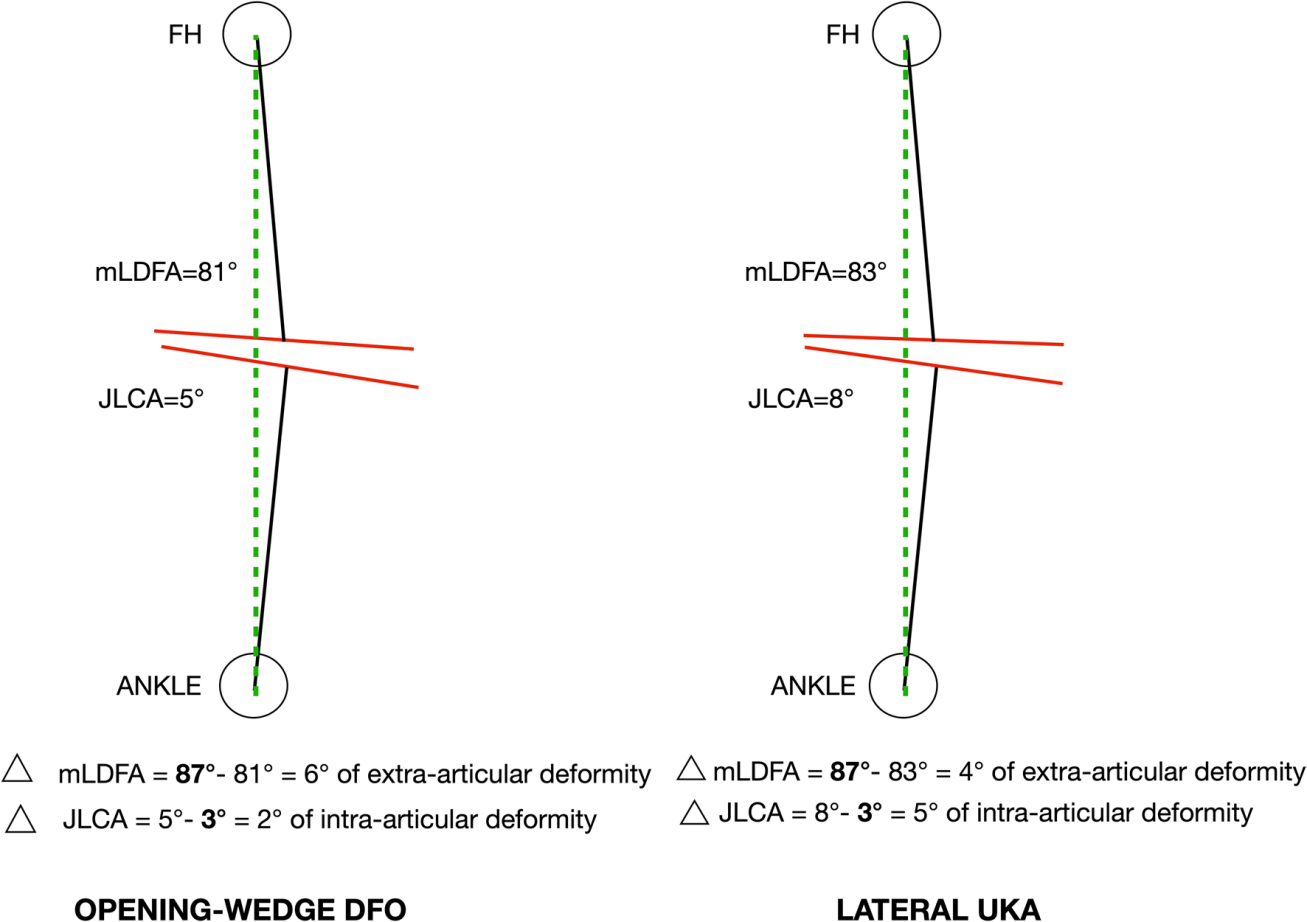
Decision process for opening-wedge DFO and lateral UKA. Δ: difference, *FH* femoral head, *mLDFA* mechanical lateral distal femoral angle, *JLCA* joint line convergence angle

The patients included in this study were characterized by a metaphyseal and intra-articular deformity assessed respectively by mLDFA and JLCA. For these reasons, the choice between DFO and UKA might be controversial, especially in active, middle-aged patients. Regarding our indication between UKA and DFO, we aimed to address the most significant deformity. Specifically, we calculated the difference between 87° and the mLDFA of the patient. If the difference was larger than the difference between JLCA and 3°, the patient was a candidate for DFO, and if the difference between JLCA and 3° was greater than the difference between mLDFA and 87°, the patient underwent UKA (Fig. 2).

### Opening-wedge distal femur osteotomy

**Fig. 3:**
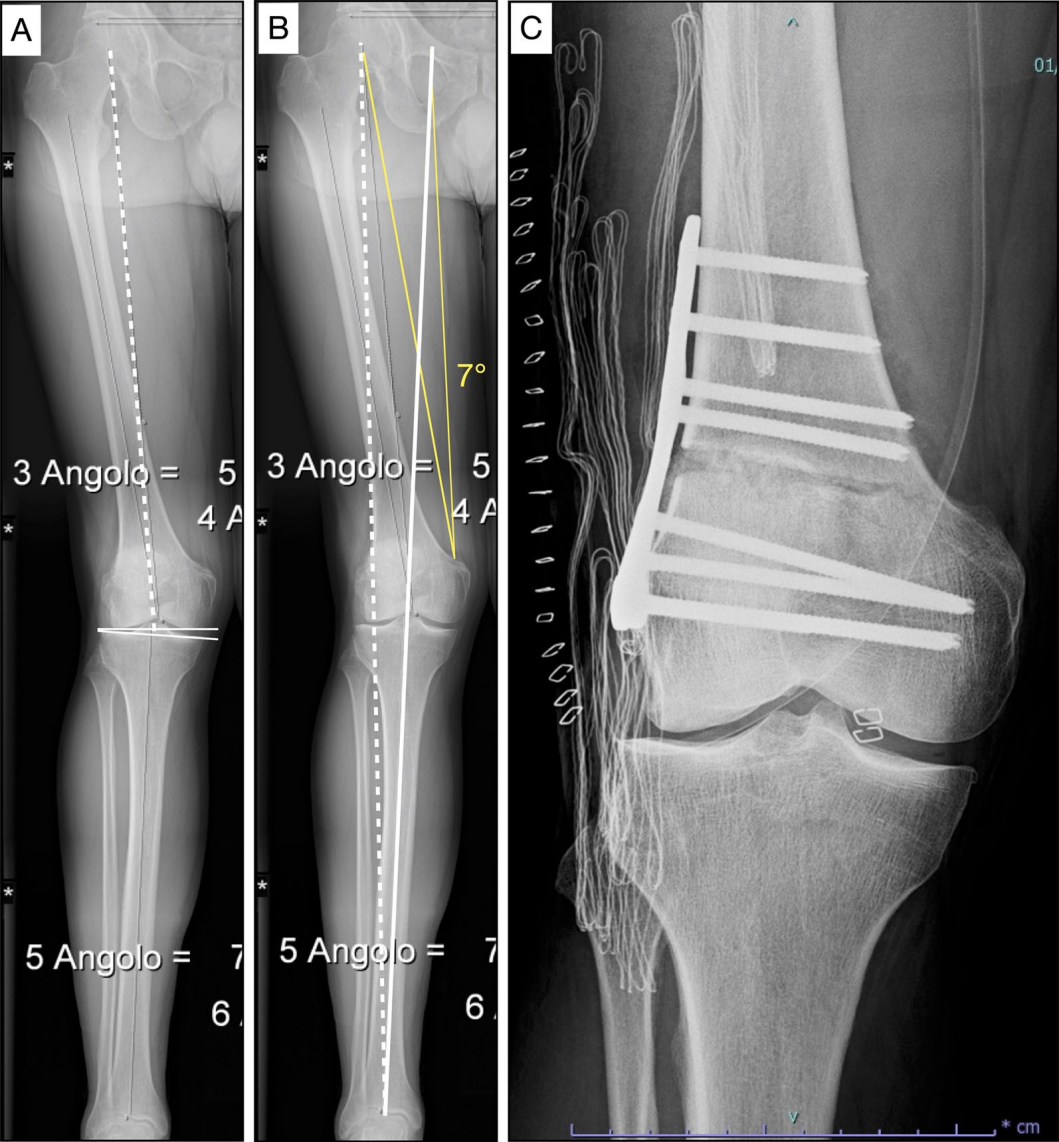
Male, 50 years old. Opening-wedge distal femoral osteotomy (DFO). **a** Preoperative long-leg standing X-rays. White dotted line: mechanical axes of the femur. White continuous lines: femur and tibia joint line. Joint line convergence angle (JLCA) = 5°, mechanical lateral distal femoral angle (mLDFA) = 84. **b** Preoperative planning of opening-wedge (DFO). Angle of correction: 7°. **c** Opening-wedge DFO with lateral locking plate (Newclip Technics, Saint Martin, France)

The operative technique included general or regional anesthesia, with the patient supine on a radiolucent table and a bump placed under the buttock to avoid external rotation of the limb. A sterile tourniquet was used. We routinely performed knee arthroscopy before the osteotomy to assess the relative integrity of the medial and patellofemoral compartment. We aimed to restore the neutral alignment of the limb through the center of the knee; the desired correction was calculated according to the Miniaci method (Fig. 3) [6]. After the incision, the starting point for the osteotomy was located under fluoroscopic control. A guide wire was drilled in an oblique direction under fluoroscopic control, and then the DFO was performed using an oscillating saw and sharp osteotomy, preserving 1 cm of the medial hinge. The osteotomy was stabilized with a locking plate and screws (Newclip Technics, Saint Martin, France). Postoperatively, the patients were required to avoid weight-bearing on the operated limb for 10 days. Partial weight-bearing with crutches was allowed for 30 days, and full weight-bearing was allowed 6 weeks after surgery. Passive and active flexion–extension of the knee was allowed immediately after surgery. We did not routinely use bone allograft, bone autograft, or synthetic bone substitution to fill the gap in opening-wedge osteotomy. However, we carefully performed small multiple incisions on the cancellous bone within the gap by sharp osteotomy in order to improve bone healing [17].

### Lateral unicompartmental knee arthroplasty

**Fig. 4:**
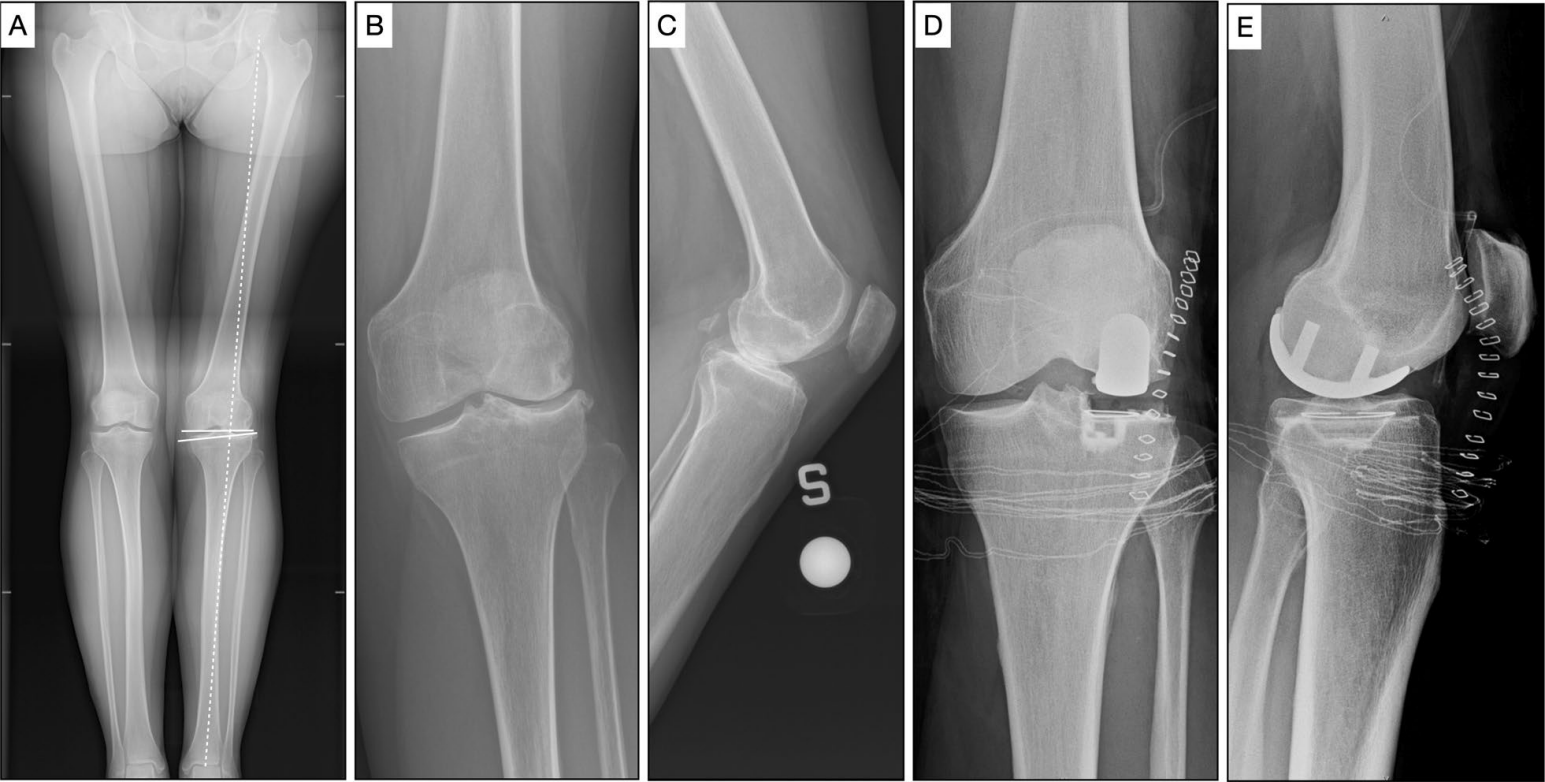
Female, 58 years old. Lateral unicompartmental knee arthroplasty (UKA). **a** Preoperative long-leg standing X-rays. White dotted line: mechanical axes of the lower limb. White continuous lines: femur and tibia joint line. Joint line convergence angle (JLCA) = 6°, mechanical lateral distal femoral angle (mLDFA) = 85. **b**, **c** Preoperative weight-bearing X-rays showed a lateral knee OA (KL3). **d**, **e** Postoperative X-rays showed a lateral UKA (LINK Unicondylar Sled Prosthesis, Waldemar Link GmbH & Co, Hamburg, Germany)

A fixed-bearing cemented prosthesis with an all-poly tibial component (LINK Unicondylar Sled Prosthesis, Waldemar Link GmbH & Co, Hamburg, Germany) was implanted (Fig. 4). All procedures were performed via a lateral parapatellar approach. Tourniquet was applied in each patient. All patients underwent lateral UKA as a separate procedure. Continuous passive knee motion was started within 24 h after surgery. Patients began progressive weight-bearing the day after surgery.

### Statistical analysis

We first performed an a priori power analysis to determine the appropriate sample size for our study. The primary study question was whether there were any differences in outcomes between the DFO and lateral UKA groups. To date, no studies have defined the minimum clinically important difference (MCID) based on KOOS and OKS in patients undergoing lateral UKA and DFO. Therefore, according to published literature, we defined the MCID as 5 and 15 points in OKS and KOOS scores, respectively [18, 19]. Hence, considering an α level with *p* = 0.05, a power of 80%, and an effect size of 0.5, it was estimated that 27 subjects each would be needed in the experimental and the control groups in order to detect a statistically significant difference in OKS and KOOS scores.

The sample size calculation was performed using G*Power software (version 3.1, Düsseldorf, Germany). All continuous variables are expressed as mean ± standard deviation (SD). Categorical variables are expressed as number and percentage. The Student *t*-test for paired data was performed for each continuous variable to compare the preoperative and postoperative values between the two groups. Differences between categorical variables were evaluated with the chi-square test. SPSS (version 17.0) statistical software was used for biometric analysis. Post hoc power analysis was performed.

### Propensity score matching

Propensity score matching is a statistical technique to limit the effect of selection bias on the estimation of causal effects in retrospective cohort studies. It is intended to overcome the covariate imbalance, to enable causal estimates of treatment effects. The six covariates included in the model were age, sex, BMI, OKS and KOOS at admission, and Tegner activity level at admission. Logistic regression was used to generate propensity scores representing the probability that a patient received a UKA versus DFO based on these data. We aimed to generate pairs of UKA and DFO patients by applying nearest-neighbor matching in a 1:1 ratio. A predefined caliper width of 0.1 without case replacement was used. This resulted in 29 pairs of UKA and DFO patients.

## Results

The preoperative characteristics of the two groups are shown in Table 1. The mean follow-up was 6.23 (range 2–11) years and 6.9 (2–10) years for the DFO and UKA groups, respectively (*p* = 0.10). The DFO group and lateral UKA group consisted of 29 patients. No statistically significant differences in gender, age, BMI, length of follow-up, or limb deformity were reported between the two groups. The medial tibiofemoral and patellofemoral joints were also characterized by a low grade of osteoarthritis in both groups. Hence, the populations of the two groups were comparable for propensity score-matched analysis. In the DFO group, OKS was reported to improve from 27.51 to 38.59 (*p* < 0.05) and KOOS from 51.14 to 67.2 (*p* < 0.05). Similarly, in the UKA group, OKS improved from 26.23 to 35.43 (*p* < 0.05) and KOOS from 50.12 to 65.91 (*p* < 0.05). However, the improvement in OKS and KOOS (delta) did not differ between groups (*p* = 0.35 and *p* = 0.95). Moreover, no statistically significant differences were reported between the UKA and DFO groups in terms of baseline or follow-up KOOS values (*p* = 0.42 and *p* = 0.63) (Table 2). Similarly, no significant differences were observed for OKS at baseline (*p* = 0.40) or follow-up (*p* = 0.75) between the two groups (Table 2). No patients in the UKA group underwent revision or other knee surgeries during follow-up. Regarding complications, no infections were detected in either group. In the DFO group, no cases of nonunion or delayed union were reported. However, 40% of DFO patients underwent plate removal. One patient in each group was characterized by the progression of medial OA with Kellgren–Lawrence (KL) grade > 3. The DFO and UKA groups were characterized by similar postoperative alignment of −3.26 and −3.0, respectively. Hence, no significant difference in terms of the HKA angle was reported between the two groups postoperatively (*p* = 0.65). After statistical analysis, a post hoc analysis was performed using G*Power software (version 3.1, Düsseldorf, Germany) to assess the power of the study to detect differences between the groups, which revealed power of 0.83 and 0.81 for OKS and KOOS, respectively. This suggests that our study had sufficient power to detect a difference in terms of OKS and KOOS between the two groups.

**Table 1:** Patient characteristics

	Opening-wedge DFO group (*N* = 29 patients)	Lateral UKA group (*N* = 29 patients)	*p*-value
Mean age, years (SD)	52.4 (3.52)	53.8 (2.2)	0.11
Mean BMI, kg/m^2^ (SD)	24.2 (2.53)	25.64 (1.5)	0.11
Female gender, no. (%)	25 (86%)	25 (86%)	0.79
Follow-up (years), mean (SD)	6.23 (1.87)	6.9 (1.92)	0.10
Grade OA compartment (KL)			
Lateral	3.40 (0.55)	3.6 (0.57)	0.52
Medial	0.80 (0.63)	1.00 (0.73)	0.48
Patellofemoral joint	1.10 (0.82)	1.05 (0.75)	0.65
Analysis of deformity			
HKA	−9.51 (2.35)	−9.67 (4.23)	0.82
MPTA	91.6 (3.2)	90.5 (2.2)	0.20
mLDFA	83.5 (1.5)	84.5 (1.6)	0.52
JLCA	4.12 (1.07)	5.01 (1.2)	0.22

Data are expressed as mean and standard deviation (SD). *BMI* body mass index, *KL* Kellgren–Lawrence, *HKA* hip-knee-ankle angle, *MPTA* medial proximal tibia angle, *mLDFA* mechanical lateral distal femoral angle, *JLCA* joint line convergence angle; negative values are used for valgus alignment

**Table 2:** Outcome data for patients undergoing opening-wedge DFO versus lateral UKA for lateral unicompartmental osteoarthritis

		Opening-wedge DFO group (*N* = 29 patients)	Lateral UKA group (*N* = 29 patients)	*p*-value
OKS score mean (SD)	Pre	27.51 (6.80)	26.23 (4.41)	0.40
	Post	38.59 (8.24)	35.43 (6.8)	0.75
	Delta	11.54 (4.92)	12.82 (4.68)	0.35
KOOS score mean (SD)	Pre	51.14 (6.3)	50.12 (5.8)	0.42
	Post	67.2 (8.80)	65.91 (8.0)	0.63
	Delta	16.8 (4.69)	17.3 (7.6)	0.95
Postoperative alignment				
	HKA	−3.26 (1.02)	−3.0 (1.4)	0.65

Data are expressed as mean and standard deviation (SD). *Pre* preoperative, *Post* postoperative, *Delta* difference between postoperative and preoperative results, *HKA* hip-knee-ankle angle. Negative values are used for valgus alignment

## Discussion

The most important finding of the present study was that lateral opening-wedge DFO and lateral UKA represent a valid treatment for isolated lateral knee OA in valgus knee with intra-articular and extra-articular deformity. Therefore, we should accept the null hypothesis, because DFO offers similar clinical improvement compared with UKA in the present selected population.

However, it should be taken into account that nearly half of the patients who underwent DFO had subsequent surgery for hardware removal due to plate prominence, discomfort, or irritation over the plate [20]. While in our cohort the percentage of patients who underwent plate removal was 40%, several studies have reported rates of hardware removal even up to 72% [21].

To the best of our knowledge, we present the first comparative study examining outcomes after a medial-opening DFO and lateral UKA in the case of intra-articular and extra-articular deformity. Our results show a significant improvement in functional scores in the DFO group. Similar results have been reported in previous studies [6–8, 20, 22–25]. However, the limited number of patients, the different hardware used, and the different postoperative rehabilitation programs make it difficult to draw comparisons. The KOOS and OKS scores increased by 16.8 and 11.54 points, respectively, in the DFO group. The improvements were statistically significant and reached the MCID [18, 19]. However, it should be noted that a similar improvement was observed in the lateral UKA group, confirming that lateral UKA represents an effective treatment [3].

Nevertheless, both osteotomy and UKA may require conversion to total knee arthroplasty (TKA) [26]. Performance of TKA after DFO must consider wound problems associated with prior incisions, retained hardware with greater risk of infection, and an oblique joint line resulting in difficulties in knee balancing [28]. On the other hand, conversion of lateral UKA to TKA is associated with fewer problems related to surgical exposure and fewer technical difficulties, especially in the case of UKA resurfacing [9].

The present study highlights that both UKA and DFO are effective in deformity correction. Hence, no significant differences in terms of postoperative HKA angle were reported between groups (*p* = 0.65). We hypothesized that the lower limb realignment after lateral UKA was driven primarily by the correction of the joint line deformity (as measured by JLCA), leaving the metaphyseal deformity (measured by mLDFA) unaltered. On the other hand, in the DFO group, the lower limb realignment was due to the correction of metaphyseal deformity (as measured by mLDFA), with no effect on deformity due to JLCA. For these reasons, a slight residual valgus deformity was maintained in each group.

The present study had limitations. Firstly, the retrospective design of the research carried the risk of intrinsic selection bias. Indeed, in cases of valgus knee and advanced lateral OA, the choice between UKA and DFO is controversial. Therefore, we decided to address the more significant deformity, leaving the other unmodified. In this way, we could obtain effective correction of the deformity, minimizing the risk of overcorrection in varus that could lead to detrimental clinical results. Additionally, the data were collected prospectively, and a propensity score-matched analysis was performed.

Secondly, we included a relatively small number of patients following application of the inclusion and exclusion criteria. However, the aim of the study was to compare the clinical results and complication rates of DFO and UKA in a very selected population where the choice between preservation surgery and joint replacement would be comparable. Lastly, the post hoc power analysis revealed that the power of the present study was 0.83. Although this value could be considered acceptable for clinical study [28], the risk of type II error warrants caution in drawing conclusions.

## Conclusion

UKA and DFO represent an effective treatment in lateral knee OA with intra-articular and extra-articular deformity. Both surgical procedures were able to provide a significant and comparable clinical improvement.

## Data Availability

Available if required.
